# Preferences of Laypeople, General Dentists, and Dental Specialists Regarding the Position of Occlusal Plane and Dental Midline in Asymmetrical Faces

**DOI:** 10.1155/ijod/4232915

**Published:** 2025-07-30

**Authors:** Yasamin Babaee Hemmati, Ghazal Zahed, Mehran Falahchai

**Affiliations:** ^1^Department of Orthodontics, Dental Sciences Research Center, School of Dentistry, Guilan University of Medical Sciences, Rasht, Iran; ^2^School of Dentistry, Guilan University of Medical Sciences, Rasht, Iran; ^3^Department of Prosthodontics, Dental Sciences Research Center, School of Dentistry, Guilan University of Medical Sciences, Rasht, Iran

**Keywords:** dental midline, dental occlusion, facial symmetry, perception of esthetics

## Abstract

**Objective:** Limited studies are available regarding the position of occlusal plane and dental midline in asymmetrical faces, and most of them only evaluated the preferences of laypeople. Thus, this study aimed to assess the preferences of laypeople, general dentists, and dental specialists regarding the position of transverse occlusal plane (TOP) and dental midline in asymmetrical faces.

**Materials and Methods:** In this analytical cross-sectional study, 20 facial photographs were designed, including one photograph of a symmetrical facial model (SFM), three photographs of asymmetrical faces with a left shift of the chin and nose (asymmetrical facial model [AFM]1), canted interpupillary line (IPL; AFM2), and canted commissure line (CL; AFM3), six photographs of asymmetrical faces with different dental midline shifts to the right and left, and four photographs of asymmetrical faces with altered position of TOP relative to the IPL and CL. The photographs were rated by 334 raters using a 4-point Likert scale. Data were statistically analyzed (α = 0.05).

**Results:** According to the opinion of all raters, photographs with 1 and 2 mm of midline deviation in the same direction as the chin and nose deviation had no significant difference with AFM1 (*p*  > 0.05). According to the opinion of laypeople, general dentists, and oral and maxillofacial (OMF) surgeons, this difference was not significant for photographs with 1 mm of midline shift in the opposite direction (*p*  > 0.05). No significant difference was found in raters' preferences regarding the position of TOP in AFM2 and AFM3 (*p*  > 0.05), except for prosthodontists and general dentists who preferred parallel position of TOP to the horizon in AFM2.

**Conclusion:** Clinicians and specialists tend to tolerate mild asymmetries more than laypeople. While demographic traits had limited impact overall, professional expertise played a significant role in shaping esthetic preferences.

## 1. Introduction

Creating a beautiful smile is the main reason for seeking orthodontic treatment by many patients [1]. A number of factors are involved in creation of an ideal smile, including the anterior teeth, maxillary bone, and facial structures; however, creation of a harmony among these components may be challenging for dental clinicians [2]. Therefore, facial, dentolabial, dental, and gingival analyses are all imperative for orthodontic treatment planning [3]. The horizontal plane is an important parameter to consider in all these analyses. The transverse occlusal plane (TOP) in the frontal view should be parallel to the interpupillary line (IPL) and the commissure line (CL) to ensure facial harmony [4]. Presence of anterior occlusal plane canting is often associated with midline deviation [5]. The treatment goals should address not only the occlusal plane level, but also its canting. “Level” is defined as vertical displacement parallel to the occlusal plane while “canting” is defined as angular displacement of the occlusal plane [6, 7].

The position of dental midline is another important parameter to consider in restorative and orthodontic diagnosis and treatment planning [8]. The dental midline is defined as the contact point of the two central incisors [9]. The facial midline is determined relative to the position of the philtrum, nose, and chin. It is often attempted to coincide the dental midline with the facial midline in continuity with a hypothetical vertical line to maximize facial and smile harmony and esthetics [6, 8].

Several studies have evaluated the preferred position of the occlusal plane relative to facial references, and the preferred maxillary dental midline position [3, 10–13]. However, the majority of such studies were conducted on symmetrical faces, while, asymmetry is a common finding even in an attractive face [12]. Asymmetry can lead to canting of the midline, TOP, and IPL, as well as chin and nose deviation, and may subsequently affect the occlusion, facial esthetics, function, and intermaxillary relationship [13, 14]. Nonetheless, occlusal plane canting or dental midline deviation may require correction in an individual with a symmetrical face, while the same conditions may not be problematic, and may not require correction in a patient with an asymmetrical face. In restorative reconstructions, it is also challenging to decide on whether or not using the same landmarks and usual protocols of smile design of symmetrical faces for patients with an asymmetrical face. The limited number of studies available on asymmetrical faces [2, 9, 12, 15–17] mainly evaluated the preferences of laypeople, and studies on the preferences of general dentists and dental specialists are scarce [12]. Also, some disagreements have been reported among dentists, laypeople, and dental specialists regarding preferred smile characteristics [12, 18, 19]. This is noteworthy since dentists should be well aware of the esthetic perception of laypeople to prevent unnecessary cosmetic procedures. Moreover, many treatments require a multidisciplinary approach, and reaching a consensus in this regard would enhance communication to meet the treatment goal [18]. This study is also innovative in that it is the first to assess how clinicians' experience and the educational level of laypeople influence esthetic preferences regarding dental midline and occlusal plane position in asymmetrical faces, two demographic factors that have been largely overlooked in prior research. Thus, this study aimed to assess the preferences of laypeople, general dentists, and dental specialists regarding the position of TOP and dental midline in asymmetrical faces. The null hypotheses of the study were that chin and nose deviation would have no significant effect on the preferred position of dental midline, and IPL or CL canting would have no significant effect on the preferred position of TOP.

## 2. Materials and Methods

The population of this analytical cross-sectional study comprised of dental specialists including orthodontists, prosthodontists, and oral and maxillofacial (OMF) surgeons, general dentists, and laypeople. The study protocol was approved by the ethics committee of Guilan University of Medical Sciences (IR.GUMS.REC.1400.607).

**Figure 1:**
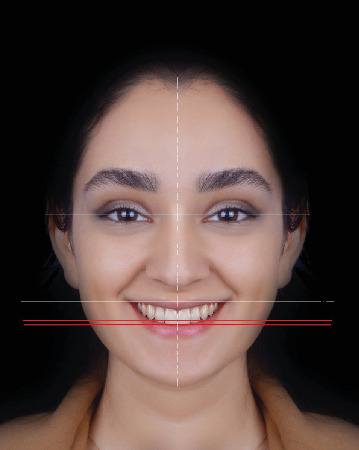
Digital SFM with superimposed facial reference lines, including the IPL, CL, facial midline, and the TOP.

A smiling facial photograph was obtained from a young female candidate after briefing her about the study objectives, and obtaining her written informed consent for publication of her photograph for research purposes. The photograph was obtained with a Canon camera (Canon, Tokyo, Japan) fixed on a tripod with 120 cm distance from the candidate [20]. The obtained photograph was precisely edited using Adobe Photoshop CS3 Extended software version 20.0.2.30 (San Jose, CA, USA). A symmetrical facial model (SFM) was digitally obtained by mirroring one side of the face (Figure 1). The photograph visualized the teeth from the maxillary second premolar of one side to the other side with <1 mm gingival show. The teeth were free from caries or restorations, the gingiva was healthy and pink in color with no inflammation, the gingival margin of the maxillary teeth was at the same level and symmetrical bilaterally, the IPL, CL, and TOP were parallel, and the dental midline coincided the facial midline [4, 21, 22].

The asymmetrical facial models (AFMs) were designed using Photoshop software. Three AFMs were evaluated in the present study; in the first one (AFM1), the nose and the chin were digitally shifted by 3 mm according to the previously reported visual detection threshold [8]. Beyer et al. [23] demonstrated that direction of facial asymmetry had no significant effect on the ratings. Thus, shifting of the dorsum, nose tip, and chin was performed only toward the left side to prevent unnecessary increase in number of images and subsequent confusion of the raters. The AFM2 model had IPL canting by 3° toward the left, and the AFM3 model had CL canting by 3° toward the left. The nose and the chin had no deviation in AFM2 and AFM3, and other components were also intact.

**Figure 2:**
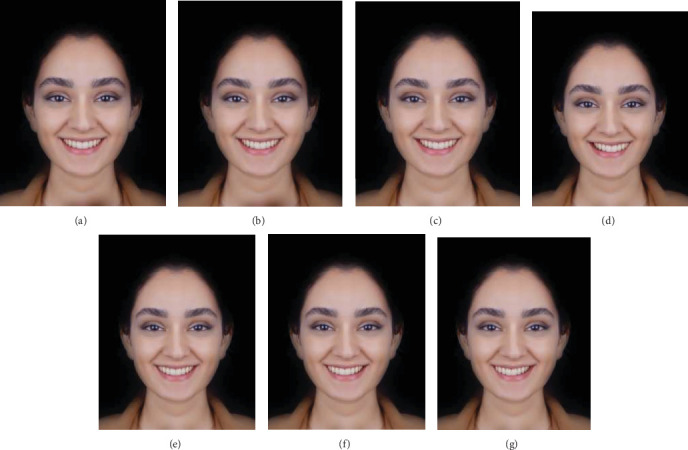
Photograph with nose and chin deviation and different positions of dental midline: (a) AFM1, (b) 1 mm shift to the left, (c) 2 mm shift to the left, (d) 3 mm shift to the left, (e) 1 mm shift to the right, (f) 2 mm shift to the right, and (g) 3 mm shift to the right.

To assess the preferred position of dental midline in a patient with a deviated nose and chin (AFM1), a progressive midline shift was applied in Photoshop software to create six different versions: DM-1L, DM-2L, and DM-3L with a midline shift toward the left by 1, 2, and 3 mm, respectively, and DM-1R, DM-2R, and DM-3R with a midline shift toward the right by 1, 2, and 3 mm, respectively (Figure 2a–g).

**Figure 3:**
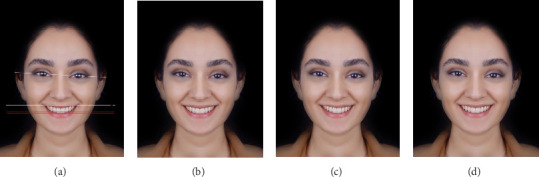
Photograph with IPL canting: (a) facial reference lines superimposed on AFM2, showing the canted IPL, TOP, and CL, (b) AFM2 without superimposed lines, (c) TOP parallel to canted IPL, and (d) TOP with a 1.5-degree deviation in the same direction as the IPL canting.

To assess the preferred position of TOP in a face with only 3° of IPL canting toward the left (AFM2), two digital images were designed with different TOP positions: TOP parallel to the canted IPL (TOP.IPL.p) and TOP with a 1.5-degree angulation in the same direction as the IPL canting (TOP.IPL.a1.5), which was in fact the bisector of the angle formed between the IPL and CL planes (Figure 3a–d).

**Figure 4:**
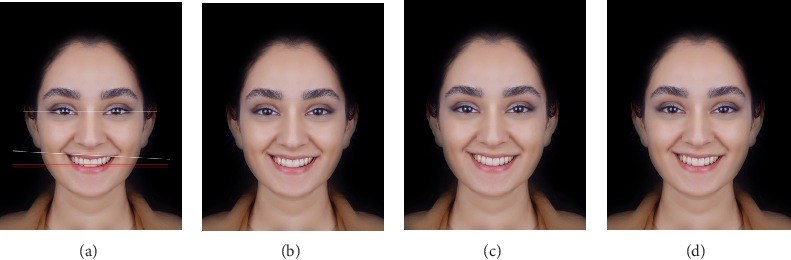
Photograph with CL canting: (a) facial reference lines superimposed on AFM2, showing the canted IPL, TOP, and CL, (b) AFM3 without superimposed lines, (c) TOP parallel to canted CL, and (d) TOP with a 1.5-degree deviation in the same direction as the CL canting.

To assess the preferred position of TOP in a patient with only a 3-degree CL canting towards the left (AFM3), two images with different CL positions were digitally designed: TOP parallel to the canted CL (TOP.CL.p) and TOP with a 1.5-degree angulation in the same direction as the CL canting (TOP.CL.a1.5), which was in fact the bisector of the angle formed between the IPL and CL planes (Figure 4a–d). The 3-degree canting for AFM2 and AFM3 models was selected according to previous studies [4, 24].

Finally, 14 photographs including one SFM, three AFMs, six photographs with different midline positions, and four photographs with different TOP positions were obtained. The final photographs were prepared with standard color and 300 dpi resolution. An online questionnaire was designed in www.survey.porsline.ir and sent to the raters. The inclusion criteria for the raters were orthodontists, prosthodontists, OMF surgeons, general dentists, and laypeople. The laypeople had to have at least high-school diploma or nondental education (dental assistants, oral hygienists, and prosthetic technicians were excluded), a minimum age of 18 years, no history of orthodontic treatment in the past 5 years, no history of artistic activities, and no dentist family member (parent, spouse, or siblings) [7, 9]. The participants who did not rate all the images, those not willing to remain in the study, pregnant women, those with cognitive or physical impairments, and raters with depression, psychological disorders, or emotional/social trauma were excluded [7, 9]. The participants received sufficient information about the objectives and protocol of the study, and were ensured about the confidentiality of their information. Those willing to participate in the study filled out a questionnaire, which included questions regarding the scoring of images, questions regarding the eligibility criteria, and questions about their demographic information including age, gender, level of education (for laypeople), and work experience since graduation (for dentists and dental specialists). The questionnaire had three separate steps for scoring of image series (AFM1, AFM2 and AFM3). The first comparison included SFM, AFM1 and six images with altered position of the dental midline. The second comparison included SFM, AFM2, and two images with different TOP positions and their correlation with AFM. The third comparison included SFM and AFM3 images and two images with different TOP positions and their correlation with AFM3. To prevent a common scoring bias of giving a lower score to the first image due to not being aware of the next images, the images of each step were shown to the raters in two modes. In the first mode, the raters could only view the images. In the second mode, the same images were shown randomly but with a different order from the first mode, and the raters were asked to rate the attractiveness of each image using a 4-point Likert scale: 4 = acceptable, 3 = almost acceptable, 2 = almost unacceptable, and 1 = unacceptable [22]. Similar to prior studies [2, 8, 13] evaluating facial esthetics, raters were not provided with a standardized definition of “attractiveness.” Instead, they were instructed to rate each image based on their own esthetic perception and clinical experience. This approach was intended to reflect the subjective nature of esthetic judgment in real-world contexts. Each image was displayed on a separate page. There was no time limit for rating of each image, and the raters could not go back to the previous image.

The Shapiro–Wilk test was used to analyze the normality of data distribution. Accordingly, repeated measures ANOVA with the Greenhouse-Geisser and Huynh–Feldt corrections and the Bonferroni test, ANOVA with Tukey post-hoc test, and the Kruskal–Wallis test were applied. The generalized estimating equation (GEE) with a continuous response was used to analyze the data according to the demographic variables. Unstructured working correlation matrix was selected with Quasi Likelihood under Independence Model Criterion. SPSS version 28 (IBM SPSS Statistics; IBM Corp., NY, USA) was used for all statistical analyses at 0.05 level of significance.

## 3. Results

A total of 334 eligible participants including 138 laypeople, 76 general dentists, 29 orthodontists, 64 prosthodontists, and 27 OMF surgeons filled out the questionnaire, and their demographic information is presented in Table 1.

In all comparisons made in the present study, the score of SFM image was significantly higher than other images (*p* < 0.05). A significant difference was found in the preferences of all rater groups regarding the position of dental midline in presence of nose and chin deviation (AFM1) (*p* < 0.001). All rater groups gave the highest score to SFM followed by AFM1. DM-1L, DM-2L, and DM-1R images had no significant difference with AFM1 in the groups of laypeople, general dentists, and OMF surgeons. DM-1L and DM-2L had no significant difference with AFM1 in the groups of orthodontists and prosthodontists (*p* > 0.05, Table 2). Statistically significant differences were observed in the mean esthetic scores among the five rater groups for the following images (Table 2): DM-1L (*p*=0.031), DM-2L (*p*=0.044), DM-3L (*p* < 0.001), DM-2R (*p* < 0.001), DM-3R (*p* < 0.001), and DM-a7.5R (*p* < 0.001).

Regarding the preferred position of TOP in presence of canted IPL (AFM2), no significant difference was found between AFM2 and TOP.IPL.p and TOP.IPL.a1.5 according to the opinion of laypeople, orthodontists, and OMF surgeons (*p*  > 0.05). Nonetheless, general dentists and prosthodontists gave the highest score to AFM2 and the lowest score to TOP.IPL.p. In both the aforementioned rater groups, a significant difference existed in the ratings of AFM2 and TOP.IPL.p (*p*=0.002). In the group of prosthodontists, a significant difference existed in the ratings of TOP.IPL.p and TOP.IPL.a1.5 (*p* < 0.05, Table 3). Statistically significant differences were observed in the mean esthetic ratings among the five rater groups for AFM2 (*p*=0.009) and TOP.IPL.p (*p*=0.016).

**Figure 5:**
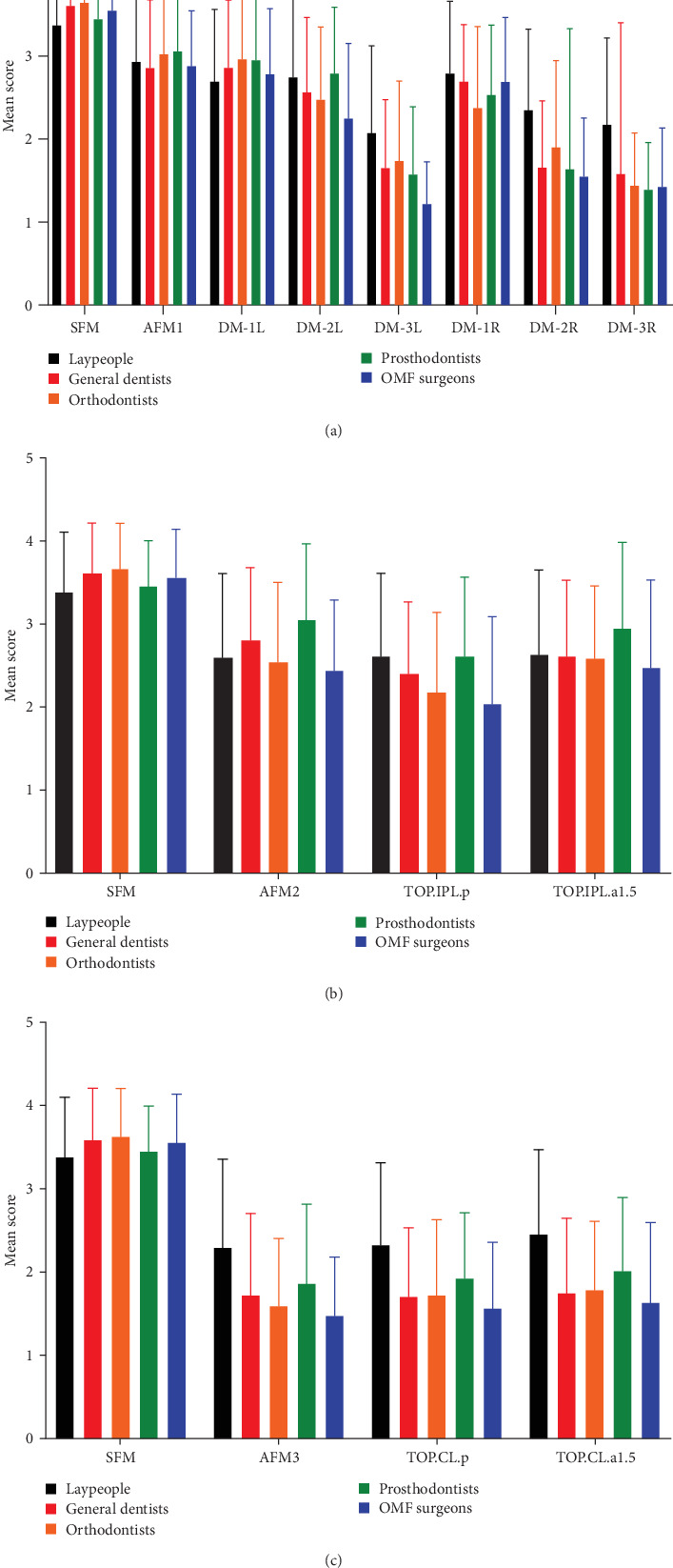
Comparison of mean esthetic scores across different rater groups for various conditions: (a) midline deviation in the presence of nose and chin deviation (AFM1), (b) occlusal plane positioning in the presence of IPL canting (AFM2), and (c) occlusal plane positioning in the presence of commissural line canting (AFM3).

Regarding the preferred position of TOP in an individual with canted CL (AFM3), no significant difference existed between AFM3 with TOP.CL.p or TOP.CL.a1.5 in any rater group (*p* > 0.05, Table 4). Statistically significant differences were found in the mean esthetic scores among the five rater groups for AFM3, TOP.CL.p, and TOP.CL.a1.5 (*p* < 0.001).  Figure 5 presents three bar charts (a, b, and c) derived from the corresponding tables to visually summarize the esthetic ratings across different scenarios and improve clarity of interpretation.

Table 5 shows the effect of demographic factors on the preferred position of dental midline. In the photograph with nose and chin deviation (AFM1), demographic factors had no significant effect on the preferred position of dental midline (*p* > 0.05). Nonetheless, age of orthodontists and prosthodontists and the work experience of orthodontists had a significant impact on the preferred position of dental midline (*p* < 0.05). Level of education of laypeople had a significant effect on their ratings for the preferred position of TOP in the photograph with IPL canting (AFM2) (*p*=0.003). However, the effects of gender (*p*=0.755) and age (*p*=0.548) were not significant. Moreover, age of general dentists and OMF surgeons, and age, gender, and work experience of prosthodontists had a significant effect on this parameter (*p* < 0.05). Nonetheless, none of the demographic factors of orthodontists had a significant effect on this parameter (*p* > 0.05). Demographic characteristics had no significant effect on the ratings of the rater groups regarding the preferred position of TOP in presence of CL canting (AFM3) (*p* > 0.05), and only age of prosthodontists was a significantly influential factor in this regard (*p*=0.017).

## 4. Discussion

It is the dentist's responsibility to inform the patient about the significant and insignificant changes that occur in the course of treatment, and then allow the patient to make the final decision. Understanding the parameters that cause an unesthetic appearance and are noticed by the laypeople, and their association with the parameters noticed by dental clinicians can aid in appropriate restorative treatment planning. According to the obtained results, the null hypotheses of the study were rejected.

In the present study, a Likert scale was used instead of a visual analog scale to decrease subjectivity in the scoring process and enable a more accurate appraisal [8]. The subjective nature of facial esthetics poses an inherent challenge in studies of this kind. In line with most previous research [2, 8, 25] on esthetic perception, we intentionally refrained from providing a standardized definition for “attractiveness,” allowing the participants to rely on their own clinical judgment or intuitive assessment. While this approach enhances ecological validity—by simulating real-life patient evaluations—it may also introduce variability, particularly across individuals from different professional, cultural, or educational backgrounds. This subjectivity might have contributed to the subtle differences observed among the rater groups, especially in borderline cases. Nevertheless, the emergence of consistent patterns (e.g., preference for symmetry and certain midline shifts) suggests that shared perceptual norms may still exist across diverse rater populations.

According to the present results, in all comparisons of all three AFM models, the symmetrical photograph acquired the highest score in all three rater groups, compared with other photographs, which was in agreement with previous findings [9, 12, 16]. The majority of previous studies on the preferred position of dental midline evaluated symmetrical faces while clinicians commonly encounter some degrees of facial asymmetry in the clinical setting [16]. Of different asymmetries, nose and chin deviation is the most common in patients, which can interfere with the position of dental midline [11]. According to the present results, in an asymmetrical face, photographs with a central midline and 1–2 mm of midline deviation in the same direction as the nose and chin deviation, and 1 mm in the opposite direction equally acquired the highest score in the groups of laypeople, general dentists, and OMF surgeons; also, only the abovementioned photographs were esthetically acceptable (score >2) in the groups of general dentists and OMF surgeons; however, all photographs were esthetically acceptable according to the opinion of laypeople. In the groups of orthodontists and prosthodontists, photographs of central midline and 1 and 2 mm of midline deviation in the same direction as the asymmetry equally acquired the highest score, and were esthetically acceptable (score >2). In a study by Silva et al. [8] on the laypeople, the detection threshold was 2 mm when the midline shift was in the same direction as the chin and nose deviation, and 1 mm when it was in the opposite direction. These results also align with the findings of studies that demonstrated that visual harmony rather than rigid symmetry often governs perceived facial esthetics [8, 26]. This supports the concept of the “facial flow curve” described by Silva et al. [27] in which midline alignment with the curve enhances esthetic acceptance. Koseoglu et al. [9] reported that when the dental midline coincided with the direction of the facial flow curve—referred to as the “green side”—it was perceived as more esthetically pleasing compared to when the midline deviated toward the opposite or “red side” of the curve. Also, Kuruhan and Çoban Büyükbayraktar [25] demonstrated that the acceptable threshold for dental midline shift in a symmetrical face was 4 mm for laypeople and 2 mm for general dentists and specialists. Moreover, they reported that in cases of mandibular asymmetry, a coinciding shift of the maxillary dental midline may still be perceived as esthetically acceptable.

Silva et al. [2] discussed that chin and nose deviation had no significant effect on people's perception of occlusal plane canting, and the occlusal plane should be parallel to the horizon or nondeviated IPL as much as possible. Thus, in the present study, asymmetrical models with IPL and CL canting were used to find the preferred position of TOP in an asymmetrical face. In the photographs with IPL canting (AFM2), no significant difference was found in the opinion of laypeople, orthodontists, and OMF surgeons regarding the selection of CL, horizon, or IPL as the reference to determine the position of TOP. In photographs with CL canting (AFM3), this difference was not significant in any rater group. Nonetheless, in images with IPL canting (AFM2), a TOP parallel to the horizon was preferred the most, and a TOP parallel to the canted IPL was the least preferred by general dentists and prosthodontists. Among the few studies conducted on this topic, Soheilifar et al. [12] and Carvalho et al. [26] stated that a TOP parallel to the IPL was preferred to a TOP parallel to CL from the perspective of laypeople, orthodontists, and OMF surgeons. In their study, asymmetry only included CL canting, and since IPL is parallel to the horizon, the preference of IPL to CL is a matter of question. Moreover, their study had some methodological differences with the present study as well as some limitations that were addressed in the current study as much as possible [28].

In the present study, demographic factors generally showed limited influence on the preferred position of the dental midline and occlusal plane; however, the effects of specific subgroup characteristics—such as age and clinical experience of professionals and educational level of laypeople—were statistically significant under certain conditions. These findings suggest that while the overall effect is minimal, individual traits may subtly shape esthetic preferences.

Only a few previous studies have systematically evaluated demographic variables such as age and gender in similar contexts, and their findings remain inconclusive [8, 26]. For instance, Silva et al. [8] and Carvalho et al. [26] reported that neither age nor gender of raters significantly affected esthetic perception. Conversely, Revilla-León et al. [13] found that older participants and male raters tended to assign higher esthetic scores. Similarly, Martins et al. [17] reported minor yet statistically significant effects for age and gender, aligning with the current findings. Some studies conducted in the broader context of facial and smile esthetics have also investigated gender-based perceptual differences [29, 30]. Cross and Cross [29] observed that female raters gave higher esthetic scores to female models compared to male raters, while no such difference was found for male models. On the other hand, another study [30] found no significant influence of rater gender.

With regard to the impact of professional expertise on esthetic judgment, the present results are in agreement with most previous research, which has consistently highlighted the role of clinical training and specialization in shaping perceptual thresholds [12, 19, 25]. However, Niknam et al. [31] who investigated the effects of age, gender, and specialty of raters on esthetic perception related to gingival display, buccal corridor size, and vertical facial proportions, concluded that only gender was a significant factor, with male raters assigning higher scores. Similarly, Rakhshan et al. [32] in their study on facial forms, midline deviations, and roll angles, found that among age, gender, and professional experience, only gender had a significant effect, with male raters slightly favoring the female model.

While previous studies compared esthetic perceptions across dental specialties, they did not stratify findings based on clinical experience [12, 14]. To the best of the authors' knowledge, the present study is the first to specifically examine the effect of years of professional experience in this context. Moreover, regarding the impact of educational level of laypeople on their esthetic perception, very few studies have addressed this variable [10]. For example, Thomas et al. [10] in their study titled “the effect of axial midline angulation on dental esthetics,” concluded that level of education of nonprofessionals was not a significant influential factor.

Therefore, although some subgroup-level patterns emerged, our overall findings support the notion that demographic variables and rater expertise exert only a modest effect on esthetic preferences related to midline position and occlusal plane canting. These trends should be interpreted with caution, and future research should adopt stratified designs to validate and further explore these preliminary observations.

These findings have notable multidisciplinary relevance. In many complex esthetic cases—especially those involving asymmetry—effective treatment planning relies on collaborative input from orthodontists, prosthodontists, and OMF surgeons. The shared perception of esthetic acceptability seen among different specialist groups in this study provides a valuable basis for aligning treatment goals. Moreover, recognizing subtle differences in esthetic thresholds across disciplines may facilitate more cohesive communication and help avoid over- or under-treatment during interdisciplinary consultations. Incorporating such perception-based data into clinical protocols can enhance consistency and predictability in team-based care.

From a clinical standpoint, the findings of this study advocate for a paradigm shift in managing patients with mild facial asymmetries. In scenarios where the dental midline deviates up to 2 mm in the direction of the chin and nose deviation—or even 1 mm in the opposite direction—both laypeople and dental professionals perceived the result as esthetically acceptable. In addition, when asymmetries such as IPL or CL canting are present, most rater groups showed no strong preference regarding whether the occlusal plane should be aligned with the horizon, the canted reference line, or an intermediate position. The only exception was among prosthodontists and general dentists, who tended to favor an occlusal plane parallel to the horizon in cases with IPL canting. These results suggest that strict alignment with facial asymmetries may not be necessary in many cases, and that patient-specific considerations should guide treatment. These insights emphasize that deviations previously considered “suboptimal” might not warrant correction if they fall within the perceptual threshold of esthetic acceptability. Clinicians are often faced with the dilemma of whether to pursue aggressive correction (e.g., complex orthodontic movement or invasive prosthetic adjustments) versus a more conservative plan. The current findings provide evidence-based support for the latter, especially when treatment invasiveness outweighs esthetic gains. By recognizing the discrepancy between clinical ideals and real-world perception, dentists can avoid overtreatment, reduce iatrogenic risk, and align their approach with patient-centered care. Ultimately, treatment decisions in asymmetrical faces should consider not only morphological deviations but also esthetic acceptability thresholds perceived by different observer groups. This approach supports more balanced, minimally invasive, and interdisciplinary management in borderline esthetic cases. These conclusions can guide practitioners toward individualized treatment strategies that respect both biological cost and esthetic expectations, ultimately improving treatment satisfaction. Furthermore, integrating these perception-based insights into digital smile design protocols may help clinicians simulate and evaluate treatment alternatives more effectively in cases involving facial asymmetry.

Despite efforts to standardize the visual stimuli, this study had several inherent limitations. First, we used only a female facial model, which may not capture gender-based differences in esthetic perception. Previous research has shown that observers are generally more critical when evaluating female faces compared to male ones, potentially due to stronger societal expectations regarding female appearance [33]. This may have contributed to stricter judgment or heightened sensitivity to asymmetries in the present study. Second, all photographs were static and two-dimensional, which lacks the depth, movement, and dynamic facial expressions present in real-life interactions. This may restrict the ecological validity of the findings. Additionally, cultural, ethnic, and regional backgrounds—which were not accounted for in this study—can significantly shape esthetic preferences, as shown in previous cross-cultural research on facial attractiveness. Therefore, the results should be interpreted with caution when applied to broader or more diverse populations. Since the photograph with nose and chin deviation included simultaneous dental midline shifts, the independent effect of skeletal asymmetry could not be evaluated. Future studies using controlled, factorial designs are recommended to isolate these variables.

To further advance this line of research, future studies may benefit from expanding both the demographic and methodological scopes of evaluation. This includes exploring esthetic perceptions across a broader range of observer characteristics—such as gender, ethnicity, and cultural background—and assessing whether clinical education, age, or professional experience influence these perceptions over time. In addition, dynamic and interactive imaging techniques may more accurately replicate the way smiles are judged in real-life settings, offering greater ecological validity. Future studies should also consider using male or androgynous facial models to determine whether the gender of the subject affects perception, particularly in borderline esthetic scenarios. Such approaches could enhance the generalizability and translational relevance of perception-based esthetic studies in dentistry.

## 5. Conclusion

Considering the limitations of the present study, it may be concluded that:

1. In an asymmetrical face with nose and chin deviation, no change in the position of dental midline would be required to improve esthetics if the dental midline is vertical and at the center line. The detection threshold of laypeople for asymmetry would be 2 mm if the midline shift follows the same direction as the nose and chin deviation, and 1 mm if otherwise.
2. The raters had no specific preferences regarding an occlusal plane parallel to the horizon, or the canted IPL or CL in an asymmetrical face. Thus, no further treatment would be required for such cases. The only exception was related to prosthodontists and general dentists who believed that in a face with IPL canting, a TOP parallel to the horizon would be esthetically more pleasant.

## Figures and Tables

**Table 1:** Demographic information of the participants.

Group	Number	Age (years)	Gender		Work experience			
		Mean and std. deviation	Male (*n*)	Female (*n*)	0–2 (*n*)	2–5 (*n*)	5–10 (*n*)	>10 (*n*)
Laypeople	138	12.20 ± 36.06	39	99	Not applicable			
General dentists	76	9.77 ± 40.33	40	36	6	23	21	26
Orthodontists	29	8.23 ± 36.21	10	19	3	8	12	6
Prosthodontists	64	9.24 ± 41.70	37	27	5	8	20	31
OMF surgeons	27	4.47 ± 37.22	16	11	0	5	14	8

**Table 2:** Scores given by different rater groups and their preferences regarding the position of dental midline.

Groups	Laypeople	General dentists	Orthodontists	Prosthodontists	OMF surgeons	LSD	*p* Value (*F*)
SFM	3.38 ± 0.73^a^	3.61 ± 0.61^a^	3.66 ± 0.55^a^	3.45 ± 0.55^a^	3.56 ± 0.58^a^	0.22	0.109 (7.55)^‡^
AFM1	2.93 ± 0.91^b^	2.87 ± 0.82^b^	3.03 ± 0.73^b^	3.06 ± 0.73^b^	2.89 ± 0.66^b^	0.28	0.936 (0.20)^†^
DM-1L	2.70 ± 0.87^ABb^	2.87 ± 0.81^BCb^	2.97 ± 0.78^BCb^	2.97 ± 0.78^Cb^	2.78 ± 0.80^BCb^	0.28	0.031 (2.69)^†^
DM-2L	2.75 ± 0.95^b^	2.57 ± 0.91^b^	2.48 ± 0.87^bc^	2.80 ± 0.80^bc^	2.26 ± 0.90^b^	0.31	0.044 (2.58)^†^
DM-3L	2.09 ± 1.04^ACc^	1.67 ± 0.81^Bc^	1.76 ± 0.95^ABcd^	1.58 ± 0.81^BCe^	1.22 ± 0.51^Cc^	0.31	<0.001 (27.40)^‡^
DM-1R	2.80 ± 0.87^b^	2.70 ± 0.69^b^	2.38 ± 0.98^c^	2.55 ± 0.83^c^	2.70 ± 0.77^b^	0.28	0.074 (2.15)^†^
DM-2R	2.36 ± 0.97^Ad^	1.66 ± 0.81^Bc^	1.90 ± 1.05^ABcd^	1.64 ± 1.70^Be^	1.56 ± 0.70^Bc^	0.30	<0.001 (44.50)^‡^
DM-3R	2.18 ± 1.05^Ac^	1.59 ± 1.82^Bc^	1.45 ± 0.63^Bd^	1.39 ± 0.58^Be^	1.44 ± 0.70^Bc^	0.29	<0.001 (41.81)^‡^
LSD	0.20	0.26	0.35	0.25	0.39	—	—
*p*-Value*⁣*^*∗*^	<0.001	<0.001	<0.001	<0.001	<0.001	—	—

*Note*: Values with different lowercase letters in the same column and values with different uppercase letters in the same row are significantly different (*p*  < 0.05). 1, 2, 3, shift of dental midline in millimeters.

Abbreviations: AFM, asymmetrical facial model; DM, dental midline; LSD, least significant difference; R/L, right/left; SFM, symmetrical facial model.

*⁣* ^*∗*^Repeated measures with Bonferroni.

^†^ANOVA.

^‡^Kruskal–Wallis.

**Table 3:** Scores given by different rater groups and their preferences regarding the position of TOP in presence of IPL canting.

Groups	Laypeople	General dentists	Orthodontists	Prosthodontists	OMF surgeons	LSD	*p*-Value (*F*)
SFM	3.38 ± 0.73^a^	3.61 ± 0.61^a^	3.66 ± 0.55^a^	3.45 ± 0.55^a^	3.56 ± 0.58^a^	0.22	0.109 (7.55)^†^
AFM2	2.59 ± 1.02^ACb^	2.80 ± 0.88^ABb^	2.55 ± 0.95^ABb^	3.05 ± 0.92^Bb^	2.44 ± 0.85^ACb^	0.32	0.009 (3.46)^‡^
TOP.IPL.p	2.60 ± 1.01^Ab^	2.39 ± 0.88^ABc^	2.17 ± 0.97^ABb^	2.61 ± 0.95^ABc^	2.04 ± 1.05^Bb^	0.33	0.016 (3.08)^‡^
TOP.IPL.a1.5	2.63 ± 1.02^b^	2.62 ± 0.91^bc^	2.59 ± 0.87^b^	2.95 ± 1.03^b^	2.48 ± 1.05^b^	0.34	0.145 (1.72)^‡^
LSD	0.17	0.23	0.34	0.25	0.37	—	—
*p*-Value (*F*)*⁣*^*∗*^	<0.001 (39.36)	<0.001 (41.98)	<0.001 (27.76)	<0.001 (15.27)	<0.001 (27.44)	—	—

*Note*: Values with different lowercase letters in the same column and values with different uppercase letters in the same row are significantly different (*p*  < 0.05). TOP.IPL.a1.5, TOP is bisector of the angle formed between the IPL and CL planes; TOP.IPL.p, transverse occlusal plane parallel to the interpupillary line.

Abbreviations: AFM, asymmetrical facial model; LSD, least significant difference; SFM, symmetrical facial model.

*⁣* ^*∗*^Repeated measures with Bonferroni.

^†^ANOVA.

^‡^Kruskal–Wallis.

**Table 4:** Scores given by different rater groups and their preferences regarding the position of TOP in presence of CL canting.

Groups	Laypeople	General dentists	Orthodontists	Prosthodontists	OMF surgeons	LSD	*p*-Value (*F*)^†^
SFM	3.38 ± 0.73^a^	3.61 ± 0.61^a^	3.66 ± 0.55^a^	3.45 ± 0.55^a^	3.56 ± 0.58^a^	0.22	0.109 (7.55)
AFM3	2.29 ± 1.07^Ab^	1.72 ± 0.99^Bb^	1.59 ± 0.82^Bb^	1.86 ± 0.96^Bb^	1.48 ± 0.70^Bb^	0.33	<0.001 (29.21)
TOP.CL.p	2.33 ± 0.99^Ab^	1.71 ± 0.83^Bb^	1.72 ± 0.92^Bb^	1.92 ± 0.80^Bb^	1.56 ± 0.80^Bb^	0.31	<0.001 (31.59)
TOP.CL.a1.5	2.45 ± 1.03^Ab^	1.75 ± 0.90^Bb^	1.79 ± 0.82^Bb^	2.02 ± 0.88^Bb^	1.63 ± 0.97^Bb^	0.32	<0.001 (35.12)
LSD	0.18	0.25	0.29	0.24	0.40	—	—
*p*-Value (*F*)*⁣*^*∗*^	<0.001 (66.42)	<0.001 (140.72)	<0.001 (89.06)	<0.001 (77.24)	<0.001 (76.29)	—	—

*Note*: Values with different lowercase letters in the same column and values with different uppercase letters in the same row are significantly different (*p*  < 0.05). TOP.CL.a1.5, TOP is bisector of the angle formed between the IPL and CL planes; TOP.CL.p, transverse occlusal plane parallel to the commissure line.

Abbreviations: AFM, asymmetrical facial model; LSD, least significant difference; SFM, symmetrical facial model.

*⁣* ^*∗*^Repeated measures with Bonferroni.

^†^Kruskal–Wallis.

**Table 5:** Effect of demographic factors on preferences of different rater groups.

Groups	Laypeople			General dentists			Orthodontists				Prosthodontists			OMF surgeons		
	AFM1	AFM2	AFM3	AFM1	AFM2	AFM3	AFM1	AFM2	AFM3	AFM1		AFM2	AFM3	AFM1	AFM2	AFM3
Gender (*p*-value)	0.414	0.755	0.231	0.887	0.317	0.685	0.056	0.570	0.817	0.397		0.033	0.304	0.509	0.203	0.094
Age (*p*-value)	0.627	0.548	0.590	0.758	0.009	0.322	0.001	0.140	0.521	0.019		0.005	0.017	0.776	0.023	0.740
Work experience (*p*-value)	NA	NA	NA	0.394	0.074	0.232	<0.001	0.651	0.612	0.401		0.010	0.224	0.816	0.594	0.406
Level of education (*p*-value)	0.636	0.003	0.297	NA	NA	NA	NA	NA	NA	NA		NA	NA	NA	NA	NA

*Note*: Generalized estimating equation.

Abbreviations: AFM, asymmetrical facial model; NA, not applicable.

## Data Availability

The data that support the findings of this study are available upon request from the corresponding author. The data are not publicly available due to privacy or ethical restrictions.
